# Supplementary material for: Diabetes genes identified by genome-wide association studies are regulated in mice by nutritional factors in metabolically relevant tissues and by glucose concentrations in islets
Source: BMC Genet. 2013 Feb 25;14:10. doi: 10.1186/1471-2156-14-10 (PMC3664586; doi:10.1186/1471-2156-14-10)
Supplement: Additional file 1: Table S1 — Primer sequences. [file 1471-2156-14-10-S1.docx]

| **Table S1. Primer sequences.** | | |  |  |
| --- | --- | --- | --- | --- |
|  |  |  |  |  |
| **Gene** |  | **Forward primer** |  | **Reverse primer** |
|  |  |  |  |  |
| *Gapdh* |  | CAA CAG CAA CTC CCA CTC TTC |  | GCT GTA GCC GTA TTC ATT GTC |
| *Adam30* |  | CCT GAC AAG TGC AAT AGC CG |  | ACC GTA TCC TAC CTC TAC AC |
| *Adamts9* |  | GTC CAT CCC TGC TAC TTG AG |  | CAC AGA TCT ATG CAT GAC TCC |
| *Camk1d* |  | GGC AAA GGA GAT GTG ATG TC |  | ACC AGC AGT CCA CAG CTT TG |
| *Cdc123* |  | CGA TGA ACA GTT CCC TGA AG |  | GGA GAG TCA TCA GTG CAA TG |
| *Cdkal1* |  | AAG GGA GAA GTC TCA GGT TTG |  | GAA TAC GCA GAG CCA TGC TG |
| *Cdkn2a (Arf)* |  | GAG GAT CTT GAG AAG AGG GC |  | GTC CTC GCA GTT CGA ATC TG |
| *Cdkn2a* |  | CGA ACT CTT TCG GTC GTA CC |  | GTC CTC GCA GTT CGA ATC TG |
| *Cdkn2b* |  | AGA TCC CAA CGC CCT GAA C |  | TTG GGT TCT GCT CCG TGG A |
| *Ext2* |  | CAT GAA CTG TGA AGA TAT TGC C |  | CAC TCA GGA CAC TTG AAC TTC |
| *Hhex* |  | GAG ACT CAG AAA TAC CTC TCC |  | TCC ATT TAG CTC GGC GAT TC |
| *Ide* |  | AGA GTT CCC CTC TCA GAA TG |  | CAC GCT TGA ACT CAG TCA TG |
| *Jazf1* |  | CAA TCC TCA GCT CCA TGT GC |  | GCA TGG TAC TTT ATG CCA TTC |
| *Lgr5* |  | AGT GTG GAC GAC CTT CAT AAG |  | TGA AGG GCC TTC AGG TCT TC |
| *Thada* |  | CTG AAG TCC TCA CAA GCA CC |  | TCA CTC TGC AGA AGG GTA AG |
| *Tspan8* |  | CTG ACT GTG CAA CTT ATC AGG |  | GCC AGT CCA AAA GCA ATT CC |
| *Txnip** |  | CGA GTC AAA GCC GTC AGG AT |  | TTC ATA GCG CAA GTA GTC CAA AGT |
|  |  |  |  |  |
| * From Minn et al., *Endocrinology* 2005 146:2397-2405 | | |  |  |
